# The Photocoagulation-Assisted Aesthetic Treatment of Phlebectasias of the Lips Using a Nd:YAG Laser with a Low Energy Level

**DOI:** 10.3390/jcm12062292

**Published:** 2023-03-15

**Authors:** Niccolò Giuseppe Armogida, Alessandra Valletta, Elena Calabria, Federica Canfora, Carlo Rengo, Gianrico Spagnuolo

**Affiliations:** Department of Neuroscience, Reproductive Sciences and Dentistry, University of Naples Federico II, 80131 Naples, Italy

**Keywords:** laser, Nd:YAG laser, venous lake, phlebectasias, vascular lesions, lips

## Abstract

Venous lakes (VLs) are benign malformations often localized in the lips, therefore impacting the self-confidence of patients. In the present study, the use of a Nd:YAG laser according to a defined protocol with a low level of absolute energy (4.9 J) is proposed for the treatment of VLs of the lips. A total of 47 patients with 50 labial VL were treated with a Nd:YAG in one laser session without local anesthesia. The area reduction was evaluated according to the Vlachakis criteria 7 and 30 days after the laser application. Additionally, oral discomfort was rated according to the Numeric Rating Scale (NRS) during and 24 hours after the procedure. All patients achieved complete clinical healing within 30 days after the laser application. In particular, patients with VL with a diameter ≤ 6 mm (62.1%) achieved a complete resolution after 7 days (*p*-value < 0.001). Such patients reported little or no discomfort (NRS 0 to 3) during the laser session and no discomfort after 24 hours (*p*-value < 0.001). No major complications were reported, and no recurrence was observed at 2-year follow-up. These findings suggest that Nd:YAG laser treatment could be an effective and well-tolerated approach to the aesthetic treatment of labial VL.

## 1. Introduction

Phlebectasias or venous lakes (VLs) are very common benign malformations of the superficial dermal layers first described by Bean and Walsh in 1956 and defined as a vascular ectasia that results from a dilation of pre-existing vessels [1,2]. The lips and the oral mucosa are two of the most frequently involved sites of the head and neck region, typically found in almost 50% of individuals over 40 years of age [2]. The pathogenesis of VL is linked to a non-genetic and age-dependent loss of elasticity of the venous stromal support, resulting in dilated blood-filled venous and capillary channels. Prolonged sunlight exposure and an elderly age are the main risk factors for VL development, especially in the lips [1,2,3].

Clinically, VLs appear as solitary, often multiple, soft, compressible, bluish–red papules, the color of which may change to dark blue or even black over time, with a size ranging from 2 to 10 mm in diameter. [4,5]. Histologically, venous lakes are frequently represented by venous ectasia with few interconnecting channels lying on a background of disintegrated connective tissue [6]; therefore, the diagnosis is clinical and is based on lesion compression resulting in an ischemia of the area without any need for an oral biopsy [7]. Of great importance, when affecting highly visible areas of the face, such as the lips, venous lakes can also alter the facial appearance, raising aesthetic concerns and impacting the patient’s self-confidence and self-reported psychological well-being.

As the main request for VL therapy localized at the lips is for aesthetic reasons, treatment modalities should not only aim to resolve the lesion but also to guarantee highly effective aesthetic outcomes with the least discomfort and the lowest rate of complications. In this regard, traditional therapeutic strategies, including surgical excision, cryosurgery, infrared coagulation sclerotherapy and electrocoagulation [8,9,10], have proved to be effective in the resolution of VL. However, postoperative complications such as bleeding, swelling, pain, textural changes, scarring or allergic reactions to the injected substance may also occur [11,12].

Over the last few years, laser- and light-based treatment modalities have been proposed as valid alternatives to treat VL, such as the argon laser, CO_2_ laser, pulse dye laser and neodymium-doped yttrium–aluminum–garnet (Nd:YAG) laser. Among these, the Nd:YAG laser has been demonstrated to be one of the most reliable and safest forms of non-invasive therapy for the treatment of VL of the head and neck region, with only a few minor adverse events reported [6].

However, to date, there have only been a few studies on the effectiveness of the Nd:YAG laser for the treatment of vascular lesions, all using different protocols (a wavelength ranging from 532 to 1064 nm, spot size ranging from 3.5–7 mm and laser fluence of 50–180 J/cm^2^) and all including a variety of lesions at different localizations [6].

Due to the heterogeneity of the protocols used and of the samples included in previous studies, clear and specific protocols for the treatment of VL of highly aesthetic regions such as the lips are lacking.

Therefore, the primary aim of the present study was to test the efficacy of a Nd:YAG laser used with a predefined protocol based on a low absolute energy for the definitive treatment of VL exclusively localized at the lips. The secondary objective was to analyze the oral discomfort during and after the laser application and to record any postoperative complications. 

## 2. Materials and Methods

### 2.1. Study Design and Participants

This was a retrospective study conducted between January 2019 and January 2020 at the University of Naples Federico II, Department of Neuroscience, Reproductive Sciences and Dentistry. The study was conducted according to the Helsinki Declaration, and the methods conformed with the Strengthening the Reporting of Observational Studies in Epidemiology (STROBE) guidelines for observational studies [13]. Concerning the data collection for the retrospective analysis, this was not considered a new clinical study and did not require any approval from the ethical committee of the University of Naples Federico II according to the guideline for observational study of the Italian Ministry of Health. As described in the above guideline, notification of the retrospective data collection was sent to the University of Naples Federico II–A.O.R.N. Cardarelli Ethical Committee.

Potentially eligible patients were invited to participate in the study and were consecutively enrolled. Informed consent was obtained from all patients.

Patients presenting with phlebectasias and seeking treatment were included, in accordance with the following criteria.

The inclusion criteria were: (a) either gender, (b) aged 18 years or older, (c) a diagnosis of phlebectasias based on the clinical characteristics of the lesion and the clinical criteria proposed by Del Pozo J et al. [7] and (d) phlebectasias of the upper or lower lips.

The exclusion criteria were: (a) the presence of other lip diseases such as leukoplakia, lichen planus, actinic cheilitis or other vascular lesions; (b) already treated phlebectasias; (c) patients treated with botulinum toxin or absorbable fillers of the lips in the last three months; and (d) patients unwilling to receive laser treatment.

### 2.2. Procedure

At admission, all the included patients underwent a comprehensive intra- and extraoral examination, and data on their demographic characteristics (age and gender), medical history and VL characteristics (number, size in millimeters and location) were recorded. Pictures of the VL were taken at the first consultation using a Nikon D7500 camera with a 105 mm lens. The diameter of each phlebectasia was measured with the millimetric scale on a dermatoscope, and the area was calculated. Pictures were also taken immediately after the laser session, as well as 7 days and 1 month after the session, to compare the lesion size. In all cases, the same operator (N.G.A.) used a Nd:YAG laser (1064 nm, Synchro FT Deka, M.E.L.A s.r.l., Calenzano (FI), Italy) with a non-contact technique at a distance of 2 cm from the phlebectasia surface. 

**Figure 1:**
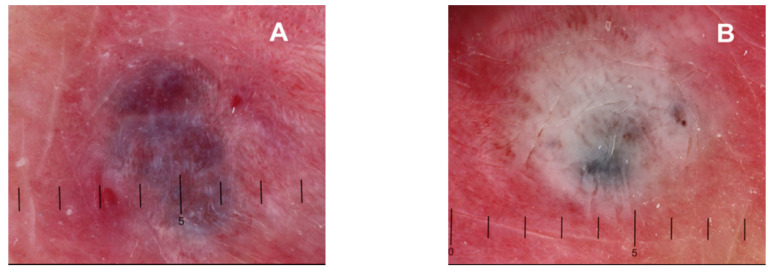
Dermatoscope view with a millimeter scale of a venous lake before the laser application (**A**) and immediately after the laser application (**B**).

The technique consisted of irradiation with a Nd:YAG laser. Laser-induced coagulation was performed in a repetitive manner with the handpiece of the irradiation delivery system held perpendicular to the skin with the lesion at its focal point. The Nd:YAG laser was set with the following operating parameters: fluence density, 100 J/cm^2^; absolute energy parameter, 4.9 J; spot size, 2.5 mm; and a single pulse frequency (quasicontinuous wave). The laser energy was delivered to all areas of the vascular lesion until the lesion turned light grey in color and achieved a hard consistency (Figure 1). In all cases, no local anesthesia was required. The patient and the operator both wore protective eye coverings.

### 2.3. Outcomes and Outcome Measures

The primary outcome was the complete healing of the VL, which was defined as a complete reduction in the lesion’s area and the absence of any eschar. The lesion’s area was measured 7 days and 1 month after the laser session, and the percentage of area reduction was calculated at each time point. The Vlachakis criteria were used to assess the area reduction as: “Excellent”, indicating a 90–100% area reduction; “Good”, a 50–89% area reduction; “Moderate”, a 20–49% area reduction; or “Poor”, a 0–19% area reduction [14].

As a secondary outcome, the pain/oral discomfort reported by the patient was evaluated immediately after the laser session and after 24 h. All patients were asked to rate their pain/discomfort using the Numeric Rating Scale (NRS-11), a validated tool for assessing pain intensity with a scale ranging from 0 to 10 (0 = no oral symptoms and 10 = the worst imaginable discomfort) [15].

Other outcomes of interest were the duration of the laser session, the presence of postoperative complications, the need for additional laser sessions and the presence of any relapses.

In case of healing with scar, the Manchester Scar Scale (MSS) was used to assess the severity of the scar (Table 1) based on the evaluation of the color, shine, contour and distortion. Possible scores range from 4 to 14, with lower scores denoting a better outcome (4 = a perfect matching of color, a matte finish, the treatment site flush with the surrounding skin and the absence of any distortion; 14 = a gross color mismatch, a shiny surface, a keloid contour and a severe distortion) [16].

The patients were evaluated 1 week, 1 month, 3 months, 6 months and 1 year after the treatment and annually thereafter.

### 2.4. Statistical Analyses

Statistical analyses were performed using SPSS software v. 23. Descriptive statistics including means, ranges, standard deviations and percentages were used to analyze the demographic and clinical characteristics of the patients and of the VL. The chi-squared test was computed to compare the percentages, and Student’s *t*-test was conducted to compare the mean scores of the variables between the time points.

As the mean diameter of the VLs was approximately 6 mm, subgroup analyses were also performed by comparing the clinical outcomes between VL ≤ 6 mm and > 6 mm. *p*-values lower than 0.05 or 0.01 were considered moderately or strongly significant, respectively.

## 3. Results

A total of 47 patients were included: 27 (57.4%) males and 20 (42.6%) females with a mean age of 63.8 years. Overall, 50 VLs were treated with the Nd:YAG laser: 48 (96.0%) localized at the lower lip and 2 (4.0%) at the upper lip. Two female patients presented a total of two and three VLs, respectively. The maximum diameter of the VLs varied from 3 to 11 mm, with a mean of 5.94 ± 1.72 mm, while the area ranged between 7 and 95 mm^2^, with a mean of 29.98 ± 17.16 mm^2^ (Table 2).

All VLs were treated according to the aforementioned protocol, with a mean operating time of the laser application of 48.44 ± 18.2 s (ranging from 30 to 120 s) (Table 3). 

Complete clinical healing was obtained in 23 VL cases (46.0%) 7 days after the laser session, while all the lesions were completely healed after 30 days (*p*-value: <0.001*). Specifically, after one week, although a complete reduction in the lesion size was achieved in all VL cases, 27 (54.0%) VLs were still characterized by the presence of eschars. Moreover, according to the Vlachakis criteria [14], the area reduction was scored as “excellent” with respect to all the VLs, with no difference after 7 and 30 days (*p*-value: 1.000).

The patients rated their oral pain/discomfort during the laser session between 0 and 3, with a mean of 1.86 ± 0.83, while no postoperative discomfort was reported 24 h after the laser session (*p*-value: <0.001**) (Table 4). 

**Figure 2:**
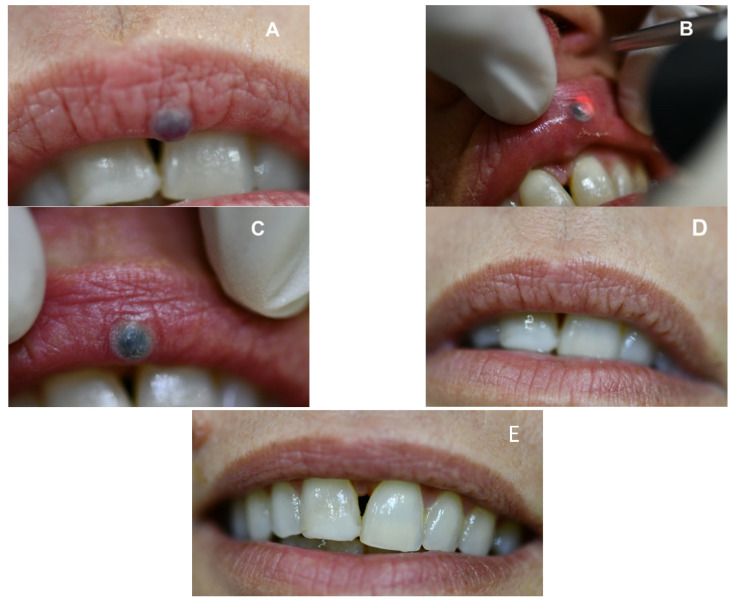
(**A**) Preoperative optical findings; (**B**) laser irradiation of the vascular lesion with no contact technique; (**C**) optical findings just after the laser application; (**D**) 1 week after the treatment; (**E**) 2 years after the treatment.

Overall, 29 (58.0%) VLs measured ≤ 6 mm, and 21 (41.0%) VLs measured more than 6 mm (Table 4). A statistically significantly higher proportion of VL ≤ 6 mm (62.1%) achieved a complete clinical healing after 7 days compared to the VLs > 6 mm (23.8%) (*p*-value: 0.007**). As expected, the mean operating time was statistically lower in the group of VLs ≤ 6 mm (*p*-value: <0.001**). Interestingly, the mean scores of oral pain/discomfort (NRS) during the laser session did not differ between the two subgroups (*p*-value = 0.711). No postoperative complications were recorded, except for the presence of a barely visible scar in one patient, with an MSS score of 7 (slight mismatch = 2, matte = 1, slightly raised = 2, mild distortion = 2). All the VLs were treated in one laser session, and none of the patients needed any further laser application. The patients were followed-up regularly, and no recurrences were observed over a minimum follow-up period of two years (Figure 2).

The proposed operative characteristics and parameters of the Nd:YAG laser protocol are displayed in Table 5 according to Hamblin M.R. (2019) [17].

## 4. Discussion

In recent years, the demand for facial aesthetic treatments has increased significantly, especially among elderly patients, resulting in clinicians being required to satisfy patient requests by proposing less invasive, more cost-effective and safer approaches [18]. New technologies such as lasers have been successfully applied in many fields of dentistry, as they provide rapid and effective treatments with little or no discomfort, thereby improving patient compliance [19,20,21].

The use of laser-assisted therapy for the management of vascular lesions has been consistently proposed in the literature. Indeed, several laser machines, such as the CO_2_ laser, the diode laser and the Er,Cr:YSGG laser, all based on invasive approaches ranging from surgical excision to photovaporization, have been widely used to treat different types of vascular lesions of the head and neck area [22,23,24,25]. Despite their proven efficacy, postoperative complications can occur, such as bleeding, swelling, textural alterations or healing with scars.

In this scenario, the non-invasive photothermal coagulation approach based on the application of a Nd:YAG laser has attracted the interest of clinicians due to its safety, manageability, effectiveness and acceptance by patients. Indeed, the use of a Nd:YAG laser allows the clinician to treat a variety of vascular lesions, such as venous malformations, hemangiomas and lymphatic malformations [26,27]. A Nd:YAG laser is a solid-state laser that uses a yttrium–aluminum–garnet crystal doped with neodymium at 1% as an active laser medium, with a semiconductor that provides the crystal with particular physical properties, emitting light energy at a wavelength of 1064 nm [26]. Moreover, Nd:YAG lasers seem to have a greater efficacy in the treatment of VL compared to other lasers [6], thanks to their wavelength pulse that has a high affinity for hemoglobin. This results in a thermal effect on the endothelium, meaning that it can penetrate about 7 mm into the tissue without interacting with the surface layers [28].

To the best of our knowledge, this is the first study evaluating the effectiveness of a Nd:YAG laser in a homogeneous sample of patients all affected by VLs localized at the lips. Other studies in the literature [6] reported the treatment of venous lakes with different body localizations and without a standardized operative protocol. Additionally, in this study, in which a Nd:YAG laser was used with the lowest laser energy according to a non-surgical transepithelial photothermal coagulation technique, the laser’s parameters were set differently than in the majority of previous studies. In the systematic review carried out by Mlacker et al. [6] the use of an argon laser [29,30,31], pulsed dye laser [22,23,24,25,32,33] and Nd:YAG laser [34,35,36] was reported. These lasers were set with a higher energy and with a shorter pulse duration, increasing the patient’s discomfort. In the present study, a Nd:YAG laser was used with an absolute low energy level of 4.9 J, a laser energy density of 100 J/cm^2^ and a spot size of 2.5 mm.

A total of 50 VLs were treated according to this procedure, which allowed for complete clinical resolution of the lesions in all cases at the 1-month follow-up, without any recurrence at the 2-year follow-up. Moreover, all the VLs, except one, healed with excellent aesthetic outcomes and no visible scars, as evaluated by MSS scores. These results are in line with those reported by Migliari et al. [34], who treated 16 lip and oral VLs with a Nd:YAG laser, achieving a complete resolution with minimal or no scarring. Migliari et al. [34] performed laser treatment under local anesthesia using a Nd:YAG in no-contact mode with a flexible quartz fiber with a diameter of 320 μm at pulse frequencies of 50 Hz for 10 s.

On the contrary, in the studies by Bekhor et al. [35] and John et al. [36] in which 34 and 31 venous lakes of the lips, cheeks and oral mucosa were treated with a Nd:YAG laser, the success rates were lower, at 94% and 87%, respectively. This could be attributable to the variability of the samples in terms of lesion size, including larger lesions and the fact that patients who had received prior treatment such as excision, cryotherapy, sclerotherapy or pulsed dye laser were also included.

In this study, the rate of clinical resolution (100%) was even higher than that reported in the study by Neumann et al. [31], in which 51 VLs of the lips were treated with an argon laser, achieving excellent cosmetic outcomes in 90% of the cases, although often requiring one to four applications before showing a clearance of the lesions. Argon lasers have the ability to emit an intense blue–green light with a peak output of 488 nm and 514 nm. The emitted pulse with the aforementioned wavelengths is absorbed by hemoglobin molecules, resulting in effective treatment of vascular lesions while avoiding the destruction of blood vessels. The differences between the results of treatment with an argon laser and a Nd:YAG laser are probably due to the reduced ability of argon lasers to penetrate the tissue compared with Nd:YAG lasers, which achieve higher penetration performance [6].

Although there is no clear evidence of the superiority of one laser machine and approach over the others in the treatment of vascular malformations including venous lakes [37], Nd:YAG lasers have been found to be more tolerable than other laser treatments [6].

In the present study, patient acceptance of the laser application was explored by recording the oral discomfort through the NRS scale. Little or no discomfort was recorded, as evidenced by the low NRS score (range 0–3), and no patient needed any topical application of local anesthetic. This result may be explained in terms of the use of a Nd:YAG laser with a low energy level, which produced less discomfort in comparison with other studies using Nd:YAG lasers with other parameters or other lasers [6].

Of note, besides the complete clinical resolution of all venous lakes, no severe or major complications, such as edema, bleeding, pain or infection, as described in the literature [6,38,39], were reported, except for one barely visible scar in one patient, demonstrating the safety of this procedure. While recurrences were reported in the other studies [6], no recurrences were recorded over the two-year follow-up period in the present sample of patients. This may be linked to the low energy level, which allows for targeting of the venous lakes for a longer time, enabling the clinical outcome of resolution without increasing the risk of scarring or complications.

The results of this study should be considered in light of some limitations. While the procedure proved to be effective for venous lakes of the lips, the effectiveness of the treatment was not evaluated on oral mucosa or other head and neck districts. Furthermore, the proposed protocol was not compared to other laser treatments or surgical procedures, preventing determination of which is the best technique to treat venous lakes.

Future research should compare lasers used with different parameters or compare lasers and surgical techniques.

## 5. Conclusions

Overall, in the present study, Nd:YAG laser application for venous lakes of the lips was proposed as an effective treatment, achieving excellent aesthetic outcomes and proving to be well-accepted and tolerated by the patients. The proposed procedure was demonstrated to have a safe profile, as no anesthesia was needed and no postoperative complications were recorded. Moreover, it was shown to be efficacious in treating lesions <11 mm in diameter with only one application.

These results suggest that Nd:YAG lasers may be used as a valid therapeutic option for the treatment of venous lakes of the lips. Nonetheless, further research studies and clinical trials comparing different laser procedures should be carried out in order to elucidate which is the most reliable and best-accepted treatment option.

## Figures and Tables

**Table 1:** Manchester Scar Scale.

	Scar Category	Points
Color	Perfect	1
	Slight mismatch	2
	Obvious mismatch	3
	Gross mismatch	4
Shine	Matte	1
	Shiny	2
Contour	Flush with surrounding skin	1
	Slightly proud/indented	2
	Hypertrophic	3
	Keloid	4
Distortion	None	1
	Mild	2
	Moderate	3
	Severe	4

**Table 2:** Demographic and clinical characteristics of the patients at baseline.

**No of patients**	47 (100%)
-Male	27 (57.44%)
-Female	20 (42.56%)
**Age (mean ± SD)**	63.82 ± 9.24 y
**No of Venous lakes**	50 (100%)
-Upper lip	48 (96%)
-Lower lip	2 (4%)
**Diameter in mm (mean ± SD)**	5.94 ± 1.72
**Area in mm^2^ (mean ± SD)**	29.98 ± 17.16

**Abbreviations:** mm = millimeters; SD = standard deviation.

**Table 3:** Comparison of clinical outcomes 7 and 30 days after Nd:YAG laser treatments.

Clinical Outcomes	After 7 Days No. of VLs (%)	After 30 Days No. of VLs (%)	*p*-Value
**Complete clinical healing**	23 (46.0)	50 (100)	<0.001 **
**Area reduction ^a^**			1.000
-Excellent	50 (100)	50 (100)	
-Good	0 (0)	0 (0)	
-Moderate	0 (0)	0 (0)	
-Poor	0 (0)	0 (0)	
	**During the session**	**After 24 h**	
**NRS** (mean ± SD)	1.86 ± 0.83	0.0	<0.001 **
**Operative time** (seconds) (mean ± SD)	48.44 ± 18.2	/	/

^a^ Area reduction based on Vlachakis criteria. A significant difference between means was measured by Student’s *t*-test and between percentages by chi-square test. ** significant at *p* ≤ 0.01. **Abbreviations**: NRS = numeric rating scale; SD = standard deviation; VL = venous lake.

**Table 4:** Comparisons of clinical outcomes between VLs with a size of less than 6 mm and higher than 6 mm.

Clinical Outcomes	≤6 mm (29 VL)	>6 mm (21 VL)	*p*-Value
**Diameter in mm (mean ± SD)**	4.75 ± 0.98	7.57 ± 1.02	<0.001 **
**Area in mm^2^ (mean ± SD)**	18.52 ± 7.27	45.81 ± 13.94	<0.001 **
**Complete clinical healing**			
-After 7 days	18 (62.1)	5 (23.8)	<0.007 **
-After 30 days	29 (100)	21 (100)	1.000
**Area reduction ^a^**			
-Excellent	29 (100)	21 (100)	1.000
**NRS (mean ± SD)**			
-During the session	1.86 ± 0.83	1.95 ± 0.86	0.711
**Operative time** (seconds) (mean ± SD)	38.03 ± 6.77	56.90 ± 8.37	<0.001 **

^a^ Area reduction based on Vlachakis criteria. A significant difference between means was measured by Students *t*-test and between percentages by chi-square test. ** significant at *p* ≤ 0.01. **Abbreviations**: mm = millimeters; NRS = numeric rating scale; SD = standard deviation; VL = venous lakes.

**Table 5:** Characteristics of the Nd:YAG laser device and operative parameters of the protocol used.

**Manufacturer**	Deka, M.E.L.A s.r.l., Calenzano (FI), Italy
**Model identifier**	Synchro FT Deka
**Year produced**	2011
**Type of emitters**	Nd:YAG laser
**Wavelength (nm)**	1064 nm
**Pulse mode**	Quasicontinuous wave
**Spot size at target**	2.5 mm
**Fluence density (J/cm^2^)**	100 J/cm^2^
**Radiant energy (J)**	4.9 J
**Application technique**	Non-contact technique at a distance of 2 cm
**Mean area irradiated**	29.98 ± 17.16
**Mean duration of laser session**	48.44 ± 18.2 sec
**Number and frequency of treatment sessions**	Single laser session

## Data Availability

Not applicable.
